# Diabetic Retinopathy: Are lncRNAs New Molecular Players and Targets?

**DOI:** 10.3390/antiox11102021

**Published:** 2022-10-12

**Authors:** Simona Cataldi, Mariagiovanna Tramontano, Valerio Costa, Marianna Aprile, Alfredo Ciccodicola

**Affiliations:** 1 Institute of Genetics and Biophysics “Adriano Buzzati-Traverso”, CNR, Via P. Castellino 111, 80131 Naples, Italy; 2 Department Experimental Medicine, University of Campania “Luigi Vanvitelli”, 80138 Naples, Italy; 3 Department of Science and Technology, University of Naples “Parthenope”, 80143 Naples, Italy

**Keywords:** diabetic retinopathy, diabetes, epigenetics, non-coding RNAs, long non-coding RNAs, competitive endogenous RNAs, gene expression deregulation

## Abstract

The growing incidence of diabetes mellitus worldwide implies the increasing prevalence of several related macro- (e.g., hypertension and atherosclerosis) and micro-vascular (e.g., nephropathy and retinopathy) complications. Notably, diabetic retinopathy (DR) is the leading cause of blindness in older diabetic patients and can occur with different degrees of severity. Chronic hyperglycemia is the main determinant of the functional damage of retinal cells. The oxidative stress, inflammatory factors and vascular endothelial growth factor signaling have been widely reported as contributors of DR onset and progression, and an emerging role has been described for different classes of non-coding RNA, including several long non-coding RNAs (lncRNAs). Here, we report the main results of all research articles (i.e., 150) listed on PubMed database from 2014 to 2022 regarding the putative role of lncRNAs in DR, including small nucleolar RNA host genes (SNHGs). Particularly, in this review we describe all lncRNAs and SNHGs with altered expression in DR and related contexts, discussing their association with DR outcomes, their mechanism of action related to DR, the molecular/functional effects, as well as the biological and experimental contexts. Thus, herein we provide an overview of the current state of knowledge regarding the putative involvement of 50 lncRNAs and SNHGs in the pathogenesis of DR, highlighting their potential as therapeutic targets or biomarkers for improving the clinical management of DR.

## 1. Introduction

In the last decade, the global prevalence of diabetes has rapidly increased, affecting to date 537 million of individuals and it is predicted to rise to approximately 783 million by 2045 [1]. Chronic hyperglycemia can lead to vascular damage, compromising the functionality of multiple tissues and organs (e.g., heart, eyes, kidneys, nerves) [2]. Accordingly, diabetic complications can be distinguished as micro- (i.e., retinopathy, neuropathy, nephropathy) and macro-vascular (i.e., coronary artery, cerebrovascular, peripheral arterial diseases) [2,3].

Notably, diabetic retinopathy (DR) represents, among the different micro-vascular complications, the most frequent with an estimated prevalence of about 35%, thus constituting the main cause of blindness. [4]. The main risk factors reported for DR are the duration of diabetes, poor metabolic control, puberty, pregnancy, hypertension, impaired blood lipid control, and kidney disease [5]. DR progression is highly variable and the non-proliferative and proliferative DR (NPDR and PDR, respectively) represent the two main clinical forms. The microaneurysms of retinal capillaries (background retinopathy) represent early clinical outcomes, whereas neovascularization (proliferative retinopathy) and macular edema arise subsequently, and they lead to vitreous or retinal detachment, and partial or total blindness [5,6].

Among the molecular factors contributing to DR onset and progression, diverse in vitro and in vivo studies have revealed that multiple factors triggering inflammation and oxidative stress—such as inflammatory cytokines, advanced glycosylation end products (AGEs) and the vascular endothelial growth factor (VEGF)—play key roles in its etiology [5,7]. Particularly, VEGF, an endothelial cell-specific mitogen, promotes angiogenesis regulating cell proliferation and retinal capillary permeability. A pivotal role of VEGF in the vascular lesions of DR has been described and its higher expression levels have been associated with increased neovascularization and vascular permeability [5]. Notably, intravitreal anti-VEGF therapies are being explored as a primary approach for the management of macular edema in DR [8]. Beyond VEGF deregulation, altered expression of several protein-coding genes (e.g., *SOD2*, *POLG1*, *NFE2L2* and those encoding Matrix Metallopeptides) and non-coding RNAs (e.g., miR-195a) has been reported in DR patients and in diabetic mouse models [9,10,11,12,13]. Particularly, increasing interest has been directed towards the role of non-coding RNAs (ncRNAs) in the regulation of key molecular pathways related to DR pathogenesis [14,15,16]. In this context, the number of studies focused on the identification and elucidation of pathophysiological roles of ncRNAs has rapidly grown thanks to the employment of NGS technologies, which have allowed for the identification, characterization and quantification of several ncRNAs in multiple disease-related context/conditions. Among them, long-non-coding RNAs (lncRNAs) turned out to be key protagonists in the gene regulation networks, able to orchestrate multiple cellular processes, such as cell proliferation, apoptosis, differentiation, cell cycle and migration [17,18,19]. LncRNAs have been generally defined as transcripts >200 nucleotides not translating proteins; however, several studies revealed that small peptides can be translated by lncRNA loci [20,21,22,23], further adding complexity to the study of lncRNAs and the effects of their deregulation. In the last decade, the key roles of lncRNAs in the regulation of gene expression have been emerging, especially by post-transcriptional and epigenetic mechanisms, including the modulation of mRNA splicing, mRNA decay, chromatin remodeling and genomic imprinting [17,24,25]. Indeed, they can act as scaffolds of protein complexes, as host genes or sponges for microRNAs, as masks of miRNA binding sites, and/or as regulators of transcription and epigenetic factors [19,26,27,28].

Although the pathogenic role of lncRNAs has been mainly described in tumorigenesis [19], more recently their involvement has emerged in multiple human pathogenic conditions, including DR. Hereafter, we report the main results obtained by reviewing all research articles collected in the PubMed database (https://pubmed.ncbi.nlm.nih.gov/, accessed on 8 October 2022) from 2014 to 2022 mentioning “diabetic retinopathy” and “long non-coding RNA” in title, entire text body or keywords [29]. Thus, also including small nucleolar RNA host genes (SNHGs), this review is focused on describing the association between the deregulation of these classes of ncRNAs and DR, providing a global overview of the main molecular/functional mechanisms and effects of deregulated lncRNAs and SNHGs in the context of DR. For instance, the interactions with key factors and signaling pathways related to DR are carefully discussed, as well as the epigenetic regulation of crucial genes and their sponging activity towards microRNAs (miRNAs). Thus, we discuss the research regarding the roles of lncRNAs described as deregulated in different in vivo and in vitro models of diabetic retinopathy, as well as in human studies, distinguishing lncRNAs whose expression has been reported as increased or reduced in this pathogenic context.

## 2. Long Non Coding RNAs with Increased Expression in Diabetic Retinopathy

Among lncRNAs reported as upregulated in DR and related contexts, the **Metastasis-Associated Lung Adenocarcinoma Transcript 1** (*MALAT1*) was the earliest lncRNA reported as being associated with DR [30]. It is located on chromosome 11q13.1 and is transcribed as a long transcript, which undergoes to 3′ end cleavage by RNase P producing *MALAT1* lncRNA and a small ncRNA known as *MALAT1*-associated small cytoplasmic RNA (mascRNA) [31]. The processed *MALAT1* lncRNA is retained in the nucleus, where it affects gene and protein expression by modulating alternative splicing, epigenetic modifications or acting as a sponge, sequestering miRNAs [32,33,34]. The over-expression of *MALAT1*, as well as genomic mutations, have been reported in different types of cancers and its role in tumor progression and metastasis has been widely described [35,36,37,38]. Interestingly, in 2014, Yan and colleagues suggested for the first time the involvement of *MALAT1* in DR [30]. Particularly, they observed a significant upregulation of *MALAT1* both in mammalian retina cells (i.e., monkey choroid-retinal endothelial cells, RF/6A) grown in high glucose (HG) conditions, and in the fibrovascular membranes and aqueous humor of diabetic patients [30]. Furthermore, the upregulation of *MALAT1* was also reported in the retina, Müller cells and primary retinal ganglion cells (RGCs) of streptozotocin (STZ)-induced diabetic models of rats [39,40], as well as in the retina of diabetic mice models [39,41]. In addition, diabetic patients—compared to healthy individuals—also display *MALAT1* overexpression, whereas reduced plasma levels were observed in glycemic-controlled patients with DR [42]. Moreover, a role of *MALAT1* in DR pathogenesis has been suggested by its knockdown in diabetic rats, which induces an improvement of retinal functions, reducing neonatal retinal vascularization and the pericytes loss, as well as capillary degeneration, microvascular leakage, and retinal inflammation [39,43]. Besides, the inhibition of retinal *MALAT1* in diabetic mice determines a recovery of the thickness of the retinal photoreceptors, reducing the diabetic neurodegeneration [41]. Notably, also in mammalian cell lines—i.e., RF/6A cells, human umbilical vein endothelial cells (HUVEC) and human retinal microvascular endothelial cells (HREC)—it reduces cell proliferation, migration, tube formation and vascular permeability [39,43,44,45]. Particularly, it has been suggested that *MALAT1* can modulate, in the retina, the progression of neurodegeneration by activating the cyclic adenosine monophosphate (cAMP)-response element binding protein (CREB) and p38 MAPK pathway. Particularly, the *MALAT1*/CREB binding is responsible for CREB phosphorylation, which leads to the inhibition of the protein phosphatase 2A (PP2A)-mediated dephosphorylation and results in a continued activation of CREB signaling [40]. Thus, *MALAT1* overexpression can regulate pathological microvascular growth, even perturbing the function of a retinal endothelial cell. Moreover, *MALAT1* interacts with the nuclear factor erythroid 2-related factor 2 (NRF2; encoded by *NFEL2* gene)—a master transcription factor involved in antioxidant processes—through the transcription regulation of kelch-like ECH-associated protein 1 (KEAP1), also suggesting that it can regulate antioxidant defense in DR [46]. Additionally, different studies reported an association of *MALAT1* with increased inflammation in DR. Indeed, it has been reported that the upregulation of *MALAT1*—in HUVECs upon hypoxic or HG conditions and in HRECs cultured in HG—is paralleled by the increase of tumor necrosis factor alpha (TNF-α), interleukin 6 (IL-6) and the serum amyloid antigen 3 (*SAA3*) [43,47,48]. Accordingly, the vitreous humors from diabetic patients revealed increased expression of *MALAT1* accompanied by increased levels of TNF-α and IL-6 [48]. Moreover, *MALAT1* knockdown in human retinal vascular endothelial cells (RVECs) grown in HG—displaying high levels of *MALAT1*, glucose-regulated protein 78 (GRP78) and C/EBP homologous protein (CHOP)—reduces both capillary morphogenesis and the inflammation, suggesting that this lncRNA can promote angiogenesis and inflammation by upregulating retinal endoplasmic reticulum stress [49,50]. Another mechanism, possibly underlying the effects of *MALAT1* on inflammatory genes expression, is the association with the master catalytic subunit of the methyltransferase polycomb repressive complex 2 (PRC2) and the consequent regulation of epigenetic mediators [48]. Moreover, *MALAT1* can exert its role in DR also acting as miRNAs’ sponge, participating in a competitive endogenous RNAs (ceRNAs) network and in turn affecting the expression of different miRNA targets. For instance, it can sponge miR-124, consequently modulating the monocyte chemoattractant protein-1 (*MCP-1*), as demonstrated in retinal microglial cells of streptozotocin-induced diabetic rats [51]. Furthermore, Shaker and colleagues [52] demonstrated an opposite expression trend of *MALAT1*, miR-20b and miR-17-3p in the serum of PDR patients. In particular, *MALAT1* levels are increased in PDR patients compared to NPDR or to healthy individuals, whereas the expression levels of miR-20b, miR-17-3p are strongly downregulated [52]. Moreover, *MALAT1* can sponge miR-125b, modulating the vascular endothelial (VE)-cadherin/β-catenin complex and the VE-cadherin 5 (*CDH5*) gene, possibly contributing to the neovascularization in DR [44]. Additionally, yes-associated protein 1 (YAP1) modulates *MALAT1* which act as a sponge of miR-200b-3p by directly binding vascular endothelial growth factor A (*VEGFA*) gene, advancing DR onset and progression [53]. In line with this finding, Yu and colleagues [45] demonstrated that *MALAT1*, *Vegfa*, and Hif-1α levels were increased in DR retinal tissues of an oxygen-induced retinopathy (OIR) mouse model, whereas the expression of miR-203a-3p was decreased [45]. Interestingly, both Müller cells and human retinal microvascular endothelial cells (HRMECs) cultured in high-glucose conditions, also display the overexpression of *MALAT1* and HIF-1α paralleled to the reduced expression of miR-320a [54]. The overexpression of miR-320a downregulates Hif-1α and inhibits invasion, angiogenesis, and vascular permeability of mouse retinal microvascular endothelial cells (MRMECs), suggesting that *MALAT1* could inhibit HIF-1α and angiogenesis by sponging miR-320a [54]. Accordingly, also HG-stimulated HRMECs display high expression of *MALAT1* and *VEGFA,* as well as a reduced expression of both miR-203a-3p and miR-205-5p [45,55]. Interestingly, the overexpression of miR-203a-3p or the knockdown of *MALAT1*—targeting miR-205-5p—suppresses the proliferation, migration and tube forming of HG-induced HRMECs, further supporting a role of this lncRNA in angiogenesis through the regulation of multiple miRNAs [45,55]. Moreover, HG exposure induces the expression of *MALAT1* and phosphodiesterase 6G (*PDE6G*)—which is involved in the phototransduction signaling cascade—also downregulating the miR-378a-3p in HRMECs [56]. *MALAT1* is able to sponge miR-378a-3p, which targets *PDE6G*, suggesting another possible mechanism underlying *MALAT1* role in DR [56]. In addition, microarray data from different rodent models of DR reveal that *MALAT1* could regulate the expression of visual perception-related genes (i.e., *Sag, Guca1a, Rho, Prph2* and *PDE6G*) by affecting miR-124-3p and miR-125b-5p [57]. Finally, *MALAT1* could influence oxidative stress, inflammation, and the capillaries’ degeneration, by affecting mitochondrial homeostasis. Indeed, *MALAT1* levels are increased in mitochondria from HRECs cultured under HG conditions, suggesting that it can also translocate from the nucleus to the mitochondria [58]. Accordingly, siRNA-mediated knockdown of *MALAT1* in HRECs reduces the alterations of the mitochondrial membrane potential and of mtDNA induced by HG concentrations [58]. Thus, in hyperglycemic conditions, the increased mitochondrial translocation of *MALAT1* can also contribute to the damage of mitochondrial structure and genomic integrity. Therefore, the targeting of this lncRNA can ameliorate multiple deleterious effects induced by hyperglycemia in retina cells.

Another lncRNA having a widely investigated putative role in DR is **Myocardial Infarction Associated Transcript** (*MIAT*), whose increased expression levels were observed in diabetic patients [59,60,61,62]. *MIAT* is an intergenic lncRNA—also known as retina non-coding RNA 2 (*RNCR2*)—mostly conserved through species and it is likely a part of the nuclear matrix [63,64]. *MIAT* is located on 22q12.1 locus associated with myocardial infarction susceptibility [63]. Interestingly, the involvement of *MIAT* lncRNA has been described in various human biological processes, such as neurogenic commitment, neuronal survival [65] and the formation of nuclear bodies [66], as well as in pathological conditions including schizophrenia [67], microvascular dysfunctions [59] and ischemic stroke [68]. Notably, in mouse models, an association between *Rncr2* expression and the retinal cell fate determination has been reported, suggesting a putative role of this lncRNA in retinal cell division regulation [64]. Interestingly, Li and colleagues [60] observed that plasma of DR patients displays a significant upregulation of *MIAT*, compared to both patients without DR and healthy individuals [60]. Similarly, further studies reported high *MIAT* levels in plasma, peripheral blood mononuclear cells (PBMCs) and retina of diabetic patients vs. healthy subjects, although *MIAT* upregulation has been also disclosed in diabetic patients without DR [59,61,62]. Accordingly, the retina of diabetic rats and Müller cells isolated from streptozotocin-induced diabetic mice, display increased levels of *MIAT* [59,69,70]. The upregulation of this lncRNA in HG conditions has been also confirmed in different mammalian primary retinal cells and cell lines, including: rat retinal Müller cells (rMC-1), retinal ganglion cells (RGC-5), human retinal pigment epithelial cells (ARPE-19), human endothelial cells (EA.hy 926), monkey chorioretinal endothelial cells (RF/6A), human umbilical vein endothelial cells (HUVECs) and human microvascular endothelial cells (HMVECs) [59,60,71]. Of note, high levels of *MIAT* in diabetic patients have been associated with coronary heart disease [42], insulin resistance, poor glycemic control, increased inflammation, and cellular senescence [61]. Interestingly, *MIAT* knockdown inhibits tube formation, migration, and proliferation in endothelial cells, and ameliorates diabetic retinal microvascular dysfunctions in vivo, also reducing apoptosis through a partial modulation of the caspase-3 expression and phosphorylation of Akt serine/threonine kinase 1 (Akt1) [59]. Moreover, increased expression of *MIAT* reduces cell viability by the activation of transforming growth factor-β1 (TGF-β1) signaling [60]. Accordingly, in diabetic rat and mouse models *MIAT* expression is positively correlated with pro-inflammatory cytokines (i.e., IL-1β and IL-6), and it also directly interacts with the nuclear factor kappa-light-chain-enhancer of activated B cells (NF-κB) and thioredoxin interacting protein (TXNIP) [69,70,71]. Accordingly, the injection of human umbilical-cord mesenchymal stem cells (HUMSCs) in the retina of diabetic rats—which induces anti-inflammatory effects—also reduces the expression levels of *MIAT*, Il-1β and Il-6, improving microvascular permeability and vascular leakage [70]. Moreover, a recent work from Yu and colleagues [72] revealed the involvement of *MIAT* in the pro-inflammatory caspase-1 (*CASP1*)-dependent cell death program, i.e., the pyroptosis, in human primary retinal pericytes (HRPCs). Particularly, both *MIAT* and *CASP1* are upregulated in HRPCs treated with advanced glycation end products of modified bovine serum albumin (AGE-BSA), whereas miR-342–3p is downregulated [72]. Interestingly, both *MIAT* knockdown and miR-342–3p mimics are sufficient to inhibit the AGE-BSA-induced pyroptosis in HRPCs, whereas miR-342–3p inhibition enhances this process [72]. Since *MIAT* binds miR-342–3p that in turn interacts with *CASP1*, this lncRNA can antagonize the inhibition of *CASP1* induced by miR-342–3p, leading to pyroptosis in HRPCs [72]. In HG-induced rat retinal Müller cells, it has been also referred that *MIAT* regulates the apoptosis program by binding miR-29b [69]. Furthermore, in silico and in vitro analyses indicated that *MIAT* affects the endothelial cell function by participating in a feedback loop with *VEGF* and miR-150-5p [59]. Thus, *MIAT* lncRNA can contribute to DR development by affecting multiple protein- and non-coding genes crucially involved in various biological processes related to retina damage.

The **Antisense Non coding RNA in the *INK4* Locus** (*ANRIL*), also known as *CDKN2B* Antisense RNA 1 (*CDKN2B-AS1*) is located in the 9p21.3 genomic region, within *CDKN2B*-*CDKN2A* gene cluster that has been reported as a susceptibility locus for cardiovascular disease [73,74], cancer [75], intracranial aneurysm [76], periodontitis [77], Alzheimer’s disease [78], endometriosis [79], glaucoma [80] and type 2 diabetes [81]. The upregulation of *ANRIL* has been reported in patients with DR compared to healthy subjects [62]. Accordingly, retina from rats with DR or STZ-induced diabetic mice display high levels of *ANRIL* [82,83] and the culture of HRECs in HG conditions is sufficient to increase *ANRIL* expression [83]. Moreover, a recent analysis reported no differences in *ANRIL* expression in serum of diabetic patients compared to healthy individuals, whereas higher levels have been detected in serum, aqueous humor, and vitreous humor of patients with NPDR and PDR, compared to both diabetic patients without DR and healthy individuals [84]. Notably, serum levels of *ANRIL* do not significantly correlate with age, but positively with diabetic duration and HbAc1 level, as well as with Ang II, p65 and *VEGF* expression in vitreous fluid of PDR patients [84]. Accordingly, *ANRIL*-knockout diabetic mice and *ANRIL*-silenced HRECs display reduced levels of *VEGF* expression [83]. Particularly, this lncRNA can regulate *VEGF* expression by binding p300, the enhancer of Zeste homolog 2 (EZH2) of the PRC2 complex and miR-200b [83]. Additionally, *ANRIL*-knockdown in retinal tissues of DR rats reduces p65 expression/phosphorylation, Bax expression and inflammatory markers (i.e., IL-1, IL-10 and MCP-1), also inducing the Bcl-2 Apoptosis Regulator (Bcl-2) protein levels and ameliorating DR pathological outcomes [82]. Thus, all these findings strongly support *ANRIL* targeting as a putative approach counteracting DR onset and progression.

The lncRNA **Nuclear Paraspeckle Assembly Transcript 1**, *alias* Nuclear Enriched Abundant Transcript 1 (*NEAT1*), is localized on the 11q13.1 chromosome and is implicated in the formation of the nuclear paraspeckles, where it interacts with various proteins forming an RNA-protein complex [85]. *NEAT1* regulates gene transcription and translation by recruiting/sequestering transcription factors or affecting mRNA splicing and protein stabilization through the interaction with RNA-binding proteins (RBPs) or miRNAs [86]. Although the first analysis of *NEAT1* in the retina of streptozotocin-induced diabetic rats revealed its downregulation [87], increased levels of this lncRNA were then detected in the retina of diabetic rats and mice, in human retinal cells (i.e., HRECs and ARPE-19) exposed to HG concentrations [88,89] and in the serum of patients with DR in contrast to healthy subjects [88]. Similarly, in vitro analyses reported contrasting results and the reduction in *NEAT1* levels was described in HG conditions both as able to induce cell apoptosis of Müller cells—by reducing brain-derived neurotrophic factor (*BDNF*)—through the upregulation of miR-497 [87] and to repress the apoptosis, increasing the proliferation of HRECs through the induction of *BCL2* apoptosis regulator (*BCL2*) and reduction of *BAX* (*BCL2* Associated X Protein) expression [88]. Moreover, *NEAT1* knockdown was also reported to inhibit the proliferation and epithelial-mesenchymal transition (EMT) of ARPE-19 exposed to HG concentrations, as well as in diabetic mice retina, through the regulation of miR-204/*SOX4* pathway [89]. The repression of *NEAT1* can exert a protective role from the hyperglycemia-induced oxidative stress and inflammation by increasing superoxide dismutase activity, reducing the levels of ROS and malondialdehyde [88], and repressing inflammatory cytokines, such as the cyclooxygenase 2 (*COX-2*), IL-6 and TNF-α [88]. Furthermore, the silencing of *NEAT1* is also able to reduce the expression of *VEGFA* and TGF-β1 induced by HG exposure in HRECs, suggesting that *NEAT1* could contribute to the development of DR modulating TGF-β1 and *VEGFA* [88]. Notably, the regulation of both *NEAT1* and *MALAT1* has been recently suggested as a putative approach for protecting the mitochondrial homeostasis, and counteracting the capillary degeneration, in DR [58].

Another well-characterized lncRNA potentially involved in DR is **HOX Transcript Antisense Intergenic RNA** (*HOTAIR*), which is localized on the chromosome 12q13.13 within the *HOXC* gene cluster, precisely between *HOXC11* and *HOXC12* genes [90]. This lncRNA is able to affect epigenetic mechanisms, modulating DNA methylation, histone modifications and nucleosome localization [91]. Interestingly, a significant increase of *HOTAIR* expression was observed in diabetic patients compared to healthy subjects [52], as well as in the serum of patients with PDR and in the retinas of diabetic animal models [92]. Moreover, HRECs exposed to hyperglycemia display high levels of *HOTAIR* and increased angiogenesis and oxidative damage, as well as mitochondrial alterations [92]. Moreover, different knockdown studies further suggested the involvement of this lncRNA in DR. Particularly, *Hotair* knockdown reduces the retinal acellular capillaries and vascular leakage in vivo [93], also inhibiting in vitro the proliferation, invasion, migration, and permeability in HG-stimulated retinal endothelial cells [93]. In addition, it has been proposed that *HOTAIR* prevents oxidative stress by the modulation of epigenetic processes and transcription factors [92]. For instance, *HOTAIR* binds the lysine demethylase 1A (LSD1), inhibiting VE-cadherin transcription and decreasing H3K4me3 levels on the promoter, also favoring HIF1α-mediated transcription of *VEGFA* [92,93].

Similarly, **KCNQ1 Opposite Strand/Antisense Transcript 1** (*KCNQ1OT1*) is reported as lncRNA modulating epigenetic modification and having a putative role in DR [94]. Also known as *KCNQ1* overlapping transcript 1 or *LIT1*, this un-spliced lncRNA is located on human chromosome 11p15.5 and is an imprinted gene at the *KCNQ1* cluster [24]. *KCNQ1OT1* displays ubiquitous expression and regulates genes crucially involved in development and in postnatal behavior [95]. Its involvement was described in various cancer types [94], as well as in eye-related diseases, including cataract development [96]. *KCNQ1OT1* participates in the proliferation and EMT of lens epithelium [97]. Notably, *KCNQ1OT1* is upregulated in aqueous humor and serum samples of DR patients compared to normal subjects, as well as in HRECs cultured in HG conditions [98]. It has been proposed that *KCNQ1OT1* could regulate DR progression, acting as a sponge of miR-1470 [98], which targets the epidermal growth factor receptor (*EGFR*) and is involved in maintaining and restoring the epithelium layer of the cornea [98,99]. In particular, *KCNQ1OT1* levels are inversely correlated to the ones of miR-1470 in individuals with DR and in HG-induced HRECs [98]. In addition, it has been demonstrated that *KCNQ1OT1* regulates HG-induced pyroptosis by sponging miR-214 [100]. In particular, *KCNQ1OT1* represses miR-214 expression with consequent upregulation of its target *CASP1* in human corneal endothelial cell line (i.e., HCEC-B4G12) [100]. The upregulation of *CASP1* results in the secretion enhancement of pro-inflammatory cytokines (e.g., IL-1β), which prompts DNA breaking and decreases in vitro migration and apoptosis, thus promoting the pathological progression of diabetic corneal endothelial keratopathy [100].

The **Hepatocellular Carcinoma Upregulated *EZH2*-Associated Long Non-Coding RNA** (*HEIH*) is a recently identified intergenic lncRNA localized on chromosomal region 5q35.3 [101]. Even though primarily identified in the cytoplasm, it localizes also in the nucleus [101]. *HEIH* was initially characterized as an oncogenic lncRNA in HBV-related hepatocellular carcinoma for its ability to inhibit cell differentiation [101]. Interestingly, *HEIH* is highly expressed both in the serum of diabetic subjects with DR and in ARPE-19 cells treated with HG concentrations [102]. Particularly, *HEIH* overexpression exacerbates cell damage, altering *VEGF* expression by the sponging of miR-939 and the activation of the *PI3K*/*AKT1* signaling pathway [102]. Moreover, *HEIH* lncRNA can significantly inhibit cell viability, induce apoptosis, promote cytochrome C release from mitochondria to cytoplasm and also to enhance caspase-3 activity [102].

**Retinal Non-Coding RNA 3** (RNCR3), also known as *MIR124-1HG* (*MIR124-1* Host Gene), is located on the chromosome 8p23.1 and has been firstly studied in diabetes-related microvascular abnormalities [103]. Interestingly, *RNCR3* is more expressed in the fibrovascular membranes of diabetic patients than in idiopathic patients [103]. Accordingly, both diabetic mice and HG-exposed RF/6A cells display increased expression of *Rncr3* lncRNA [104]. Moreover, *Rncr3* inactivation induces a decrease of retinal vascular functions in vivo, altering the *Rncr3/*Klf2/miR-185-5p interaction network [104]. Finally, *Rncr3* knockdown in diabetic mouse models inhibits reactive gliosis and reduces the cytokines release, ameliorating the viability of retinal and Müller glial cells [105]. Moreover, cell apoptosis and retinal neurodegeneration are reduced with a parallel improvement of visual function, suggesting that the targeting of this lncRNA is a promising strategy in DR [105].

***HOXA* transcript at the distal tip** (*HOTTIP*) is located on chromosome 7p15.2 and transcribed antisense to the *HOXA* gene cluster. This lncRNA has been proposed to regulate the expression of the adjacent coding genes [106]. *Hottip* was found to be upregulated in STZ-induced diabetic rats and its downregulation reduces the expression levels of inflammatory factors *ICAM-1* and *VEGF* in the retina [107]. Moreover, downregulation of *HOTTIP* significantly reduces cell viability and apoptosis in HG/H_2_O_2_-treated RF/6A cells. Finally, *HOTTIP* modulates retinal endothelial cells via the P38/MAPK signaling pathway promoting the progression of DR [107].

The **Brain-Derived Neurotrophic Factor Anti-Sense** (*BDNF-AS*) is an antisense RNA of brain-derived neurotrophic factor gene (*BDNF*), located on chromosome 11p14.1. It is involved in the regulation of *BDNF* expression, and its target genes modulate neuronal functions [108]. In the pathophysiology of DR, the formation of ischemic areas in the retina has been observed, and *BDNF-AS* is known to contribute to retinal ischemic injury of ganglion cells through the repression of *BDNF* [109]. Li and colleagues [110] observed that *BDNF-AS* and apoptosis significantly increased in ARPE-19 cells exposed to HG conditions. The downregulation of *BDNF-AS* reduces apoptosis in ARPE-19 cells under HG conditions and increased the expression of *BDNF* [110].

Blood samples from DR patients, as well as HRECs induced by HG, display increased levels also of the ***FOXF1* adjacent Non-coding Developmental Regulatory RNA** (*FENDRR*), also known as *FOXF1-AS1*, paralleled with high expression of *VEGF* [111]. Interestingly, its knockdown has been described to reduce the expression of forkhead box F1 (*FOXF1*), a transcription factor essential in the development of embryonic vascularity through the regulation of the VEGF pathway in endothelial cells [112,113].

**Figure 1:**
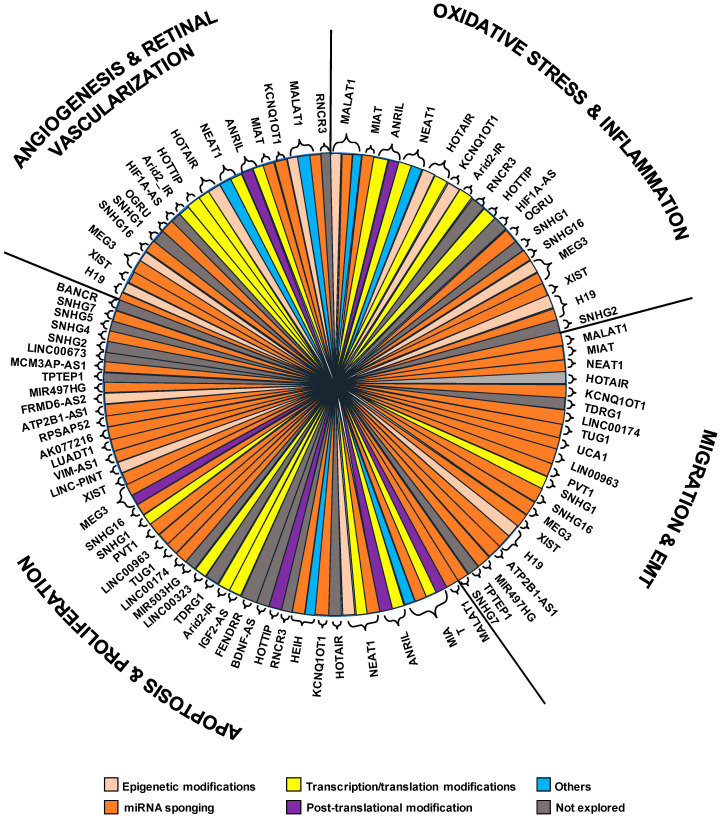
Schematic representation of molecular mechanisms through which the described lncRNAs exert their functions in different pathways/processes related to diabetic retinopathy.

However, beyond the discussed lncRNAs with increased expression in different contexts related to the DR (Figure 1, Table 1), other upregulated lncRNAs—although less characterized—have been revealed as potential contributors of DR. Particularly, in diabetic patients with DR or in human retinal endothelial cells cultured with HG concentrations, the upregulation of several different lncRNAs has been recently reported. Although further analyses are needed, for most of the mentioned lncRNAs, preliminary evidence suggests a putative role in DR. Of note, the in vitro knockdown of these lncRNAs ameliorates retinal dysfunctions improving various related-biological processes, such as proliferation, apoptosis, migration, angiogenesis, inflammation, or redox state [62,114,115,116,117,118,119,120,121,122,123,124,125,126]. For instance, ARPE-19 cells exposed to HG display increased expression of ***IGF2* Antisense RNA** (*IGF2-AS*) and enhanced apoptosis, and *IGF2-AS* silencing restrains apoptosis, inducing IGF2/Akt signaling and reducing Casp-9 expression [114]. Similarly, **AT-Rich Interaction Domain 2-IR** (*Arid2-IR*) has been reported to modulate cell apoptosis, as well as the inflammatory response and oxidative stress, by the interaction with Smad3 and the regulation of Bcl-2 and Bax protein expression [115]. Furthermore, in vitro-knockdown of **Testis Development Related Gene 1** (*TDRG1*) lncRNA restores *VEGF* expression improving cell permeability and tube formation ability of HRECs damaged by HG exposure [116]. Similarly, ***AQP4* Antisense RNA 1** (*AQP4-AS1*) in vivo-silencing reduces retinal neurodegeneration and vascular dysfunctions, counteracting retinal capillary degeneration and the reduced reactive gliosis [117]. Moreover, the silencing of the hypoxia-induced **Long Intergenic Non-Coding RNA 323** (*LINC00323*) and **miR-503 host gene** (*MIR503HG*; also known as *lnc-PLAC1-1*) in endothelial cells reduces proliferation and angiogenesis by the regulation of the angiogenic transcription factor GATA2 [118]. Finally, the increased expression of the antisense transcript of ***HIF1A* Antisense RNA 2** (*HIF1A-AS2*) in the blood of DR has been correlated to the high expression of HIFα, VEGF, MAPK, and Endogolin levels, suggesting the involvement of this lncRNA in hypoxia, oxidative stress, and angiogenesis progression through the regulation of MAPK/VEGF pathway [119]. Moreover, different over-expressed lncRNAs have been described to act as ceRNAs by sponging several miRNAs in DR-related contexts. Particularly, **Long Intergenic Non-Coding RNA 174** (*LINC00174*) is able to bind miR-150-5p, which targets the untranslated 3′ region of *VEGFA* [120], whereas **Taurine-Upregulated Gene 1** (*TUG1*) also prevents *VEGFA* suppression by the interaction with miR-145 [121]. Similarly, **Urothelial Carcinoma-Associated 1** (*UCA1*) lncRNA could sponge miR-624-3p regulating the expression of *VEGF-C* [122]. Moreover, the **Long Intergenic Non-Coding RNA 963** (*LINC00963*) may regulate the proliferation and apoptosis in HG-induced HERCs by directly targeting miR-27b [123]. Additionally, a recent work reports that the knockdown of *TUG1* ameliorates diabetic retinal vascular dysfunction through regulating miR-524-5p/*FGFR2* axis [124]. Furthermore, independent studies in patients with DR showed the negative correlations between **Plasmacytoma Variant translocation 1** (*PVT1*) and miR-128-3p, as well as between the lncRNA ***OGRU*** and miR-320, supporting that also these lncRNAs may affect miRNA-mediated networks [62,125]. Particularly, in Müller cells cultured in HG conditions, *OGRU* suppression significantly restores miR-320 expression, and it represses the ubiquitin-specific peptidase 14 (*USP14*) expression whereas, on the opposite, the upregulation of miR-320 reduces TGF-β1 signaling and impairs inflammation and oxidative stress [126].

All lncRNAs reported as upregulated in DR and the main results herein described are summarized in Figure 1 and Table 1. Overall, these studies strongly encourage further analyses aimed to assess the clinical relevance of these upregulated lncRNAs, as well as to design appropriate targeting approaches able to repress them, possibly paving the way towards the adoption of new therapeutic strategies in DR.

## 3. Long Non-Coding RNAs with Reduced Expression in Diabetic Retinopathy

Among lncRNA showing a reduced expression in DR and related contexts, the **Maternally Expressed Gene 3** (*MEG3*) is located on chromosome 14q32.2, expressed in many human tissues and initially characterized as a tumor suppressor lncRNA [127,128]. *MEG3* localizes both in the cytoplasm and nucleus [129], and multiple factors can regulate its expression, including cAMP, DNA methyltransferase family and NF-κB [127]. Recent studies highlighted a crucial role of *MEG3* in the proliferation, migration, angiogenesis, and maintenance of normal vascular endothelial cell function [130]. Interestingly, *MEG3* expression is significantly lower in the serum of diabetic patients with retinopathy compared to healthy individuals [131,132,133]. Likewise, retina and microvascular endothelial cells—exposed to HG—from DR rat models display reduced levels of *Meg3* [133,134,135]. Moreover, low levels of this lncRNA were also observed in mammalian retinal endothelial and epithelial cells (i.e., hRMECs, RF/6A and ARPE-19) cultured in HG conditions or treated with hydrogen peroxide (H_2_O_2_) mimicking diabetic stress [131,132,133,134,136]. Interestingly, in DR rats, it has been demonstrated that DNA methyltransferase 1 (Dnmt1) could promote the methylation of *Meg3* promoter, in turn reducing its expression [135]. Of note, different experiments have been carried out to evaluate the importance of *MEG3* downregulation in progression of DR. In particular, it has been observed that in vivo knockdown of *Meg3* increases retinal vessel dysfunctions, resulting in severe capillary degeneration, increased microvascular loss and inflammation [134]. Moreover, in vitro knockdown of this lncRNA promotes retinal endothelial cell proliferation, migration and neovascularization [133,134]. Conversely, overexpression of *Meg3* in STZ-induced rats reduces IL-1β expression and suppresses the endothelial mesenchymal transition, through the inhibition of the phosphatidylinositol 3-kinase (PI3K)/Akt/mTOR pathway [135,137]. Furthermore, the overexpression of *MEG3*—in ARPE-19 cells grown in HG conditions—inhibits apoptosis and inflammation, also indirectly reducing *VEGF* expression [131,132]. Particularly, *MEG3* can counteract HG-induced apoptosis and inflammation through different mechanisms, including the interaction with different miRNAs. For instance, *MEG3* can regulate *NRF2*, suppressor of cytokine signaling 6 (*SOCS6*), *NF-κB*, sirtuin 1 (*SIRT1*) and Notch1 signaling, by the modulation of miR-93, miR-34a, miR-19b, miR-204 and miR-223-3p, respectively [132,136,138,139,140]. Notably, the regulation of these signaling pathways can crucially affect DR-related processes, including the proliferation, angiogenesis and apoptosis of retinal cells [138]. For instance, it has been reported that *MEG3* downregulation negatively affects the expression of cytochrome B5 reductase 2 (*CYB5R2*) by acting as sponge for miR-6720-5p in HRMECs under HG-induced conditions [133]. Of note, CYB5R2 is involved in different oxidative reactions as well as in the regulation of angiogenesis-related genes [141,142], and its downregulation promotes angiogenesis, proliferation and migration, as well as inhibits apoptosis of HG-induced HRMECs [133]. Finally, a recent work reported that *MEG3* overexpression inhibits retinal neovascularization through the inhibition of VEGF/PI3K/AKT1 signaling pathway, further supporting the involvement of *MEG3* in angiogenesis processes [143].

Another widely studied lncRNA associated with DR is the **X Inactive Specific Transcript** (*XIST*). *XIST* was the first lncRNA identified and studied for its genomic imprinting function. It is located on the X chromosome in the q13.2 region and is able to inactivate the X chromosome in female mammal cells [144,145]. However, in recent years, it has emerged that one of the main mechanisms through which it exerts its functions is by sponging miRNAs [25]. Interestingly, two recent works reported the downregulation of *XIST* in HG-treated Müller retinal cells isolated from a diabetic mouse model, human Müller retinal cell line and ARPE-19 [146,147]. Furthermore, it has been demonstrated that the over-expression of *XIST* has a protective effect on apoptosis and migration in ARPE-19 treated with HG conditions [146], also reducing the production of pro-inflammatory cytokines in HG-treated mice and from human Müller cells [147]. However, although further analyses are still needed. This lncRNA may counteract the hyperglycemia-induced inflammation by the interaction with *SIRT1* and the induction of its expression [147]. Moreover, *XIST* over-expression produces miR-21-5p downregulation, which could in cascade determine the modulation of VEGF signaling [146].

***H19* Imprinted Maternally Expressed Transcript** is transcribed by the chromosome 11 (11p15.5) within an imprinted region close to the insulin-like growth factor 2 gene (*IGF2*) *locus*. Its localization has been reported to be mainly cytoplasmic, even though this lncRNA can localize both in cytoplasm and nucleus [148]. Interestingly, *H19* has been described to be a bi-functional RNA. Indeed, it functions either as a lncRNA or as a precursor of two conserved miRNAs encoded by exon 1 (i.e., miR-675-3p and miR-675-5p) [149]. *H19* regulates the gene expression either by the recruitment of epigenetic regulation factors to the chromatin surface or by sponging miRNAs [150]. In particular, *H19* lncRNA promotes multiple physiological processes such as inflammation, angiogenesis, apoptosis, cell death and also neurogenesis [149,151], playing a pathogenic role in different diseases, such as Beckwith-Wiedemann Syndrome and Familial Wilms Tumor [152,153] as well as many cancer types [154]. In the context of diabetes and its complications, Zhuo and colleagues [155] observed that *H19* was downregulated in STZ-induced diabetic rat models with cardiomyopathy. Later, the downregulation of *H19* was also observed in vitreous humor samples from individuals with DR, in retina of diabetic mouse models and in HG-induced HREC and ARPE-19 [17,156,157]. Conversely, Fawzy and colleagues [158] observed *H19* upregulation in the plasma of diabetic patients compared to healthy subjects, and no significant differences between patients with and without DR. However, it has been demonstrated that the over-expression of *H19* can prevent glucose-induced endothelial-mesenchymal transition (EndMT) in HRECs [17] by modulating TGF-β1 through a Smad-independent mechanism [17]. Accordingly, retinal tissue from *H19* knockout diabetic mice display the reduction of endothelial and an increase of mesenchymal markers, as well as enhanced vascular leakage [17]. Moreover, in HG-treated ARPE-19, it has been demonstrated that *H19* directly binds miR-19b that in turn increases *SIRT1* expression, which favors the reduction of the inflammatory response [157]. In a similar study, in ARPE-19 grown under HG conditions, it was observed that *H19* regulates inflammatory processes by modulation of X-box-binding protein (XBP1) expression through miR-93 inhibition [156].

The downregulation of **Long Intergenic Non-Coding RNA P53 Induced Transcript** (*LINC-PINT*) in type 2 diabetes (T2D) subjects with cardiomyopathy and/or retinopathy—but not in subjects without complications—was reported in a recent follow-up study [159]. *LINC-PINT* is located on chromosomal region 7q32.3, ubiquitously expressed and under the transcriptional regulation of p53 [160]. Interestingly, *LINC-PINT* acts as a positive regulator of cell proliferation and survival, influencing the expression of hundreds of genes, including some genes involved in p53 transcriptional network [160]. Moreover, it has been demonstrated that *LINC-PINT* interacts with PRC2 complex and is required for H3K27 trimethylation and repression [160]. Notably, the exposure of ARPE-19 and AC16 cells to HG conditions determines the downregulation of *LINC-PINT* [159]. Moreover, the overexpression and the silencing of *LINC-PINT* results in an increase or reduction, respectively, of the viability of HG-treated ARPE-19 and AC16 cells [159], suggesting that the reduction of *LINC-PINT* may favor the progression of cardiomyopathy and retinopathy in subjects with T2D [159].

Another lncRNA downregulated in T2D patients (vs. euglycemic individuals) is **Vimentin Antisense RNA 1** (*VIM-AS1*); [161]. *VIM-AS1* is located on chromosomal region 10p13 and transcribed from the opposite strand of the *VIM* gene, which is a positive regulator [162]. Interestingly, *VIM-AS1* is strongly downregulated in T2D patients with DR compared to T2D patients without complications [161]. Zeng and colleagues [161] suggested that, in the human retinal pigment epithelial cell line (i.e., H1RPE7), *VIM-AS1* interacts with miR-29, that plays a key role in HG-induced apoptosis [13,162]. Notably, miR-29 and *VIM-AS1* expression levels did not correlate in the plasma of DR patients and the over-expression of *VIM-AS1* in H1RPE7 cells did not alter miR-29 levels [161]. However, the effects of miR-29 on HG-induced apoptosis were reduced by *VIM-AS1* over-expression in h1RPE7 cells [161].

The **Lung Adenocarcinoma Associated Transcript 1** (*LUADT1*) is a known lncRNA exerting oncogenic properties in colorectal cancer and melanoma, located on 6q24.3 *locus* [163,164]. Indeed, *LUADT1* silencing induces cell cycle arrest and significantly inhibits tumor growth both in vivo and in vitro in lung adenocarcinoma [165]. Interestingly, *LUADT1* is specifically downregulated only in T2D patients with DR, suggesting that the reduced expression of this lncRNA in DR may be associated with retina lesions rather than with the hyperglycemia [166]. Moreover, prediction analysis showed that *LUADT1* lncRNA can bind miR-383 [166]. Interestingly, although *LUADT1* and miR-383 display opposite expression levels in the plasma of DR patients, no significant correlation has been demonstrated [166]. Accordingly, over-expression of *LUADT1* and miR-383 in retinal pigment epithelial cells (i.e., RPEpiC, H1RPE7) do not affect each other the expression [166]. Nevertheless, the over-expression of *LUADT1* increases the level of peroxiredoxin 3 (*PRX3*) expression, also decreasing cell apoptosis [166]. The over-expression of miR-383 exerts the opposite role on *PRX3* expression and apoptosis, suggesting that *LUADT1* may act as ceRNA of miR-383 regulating *PRX3*, and in turn improving cell apoptosis in the context of DR [166].

Furthermore, other lncRNAs have been shown to be downregulated in different contexts related to the DR (Table 2), but more investigations are still needed. In particular, low expression of ***AK077216***, **Ribosomal Protein SA Pseudogene 52** (*RPSAP52*) and ***ATP2B1* Antisense RNA 1** (*ATP2B1-AS1*) has been observed in patients with DR [167,168,169]. Interestingly, the over-expression of *AK077216* and *RPSAP52* in HG-induced ARPE-19 and RPE cells, respectively, inhibits cellular apoptosis [167,168]. The former was reported as able to downregulate miR-383 [167], the latter as able to interact with miR-365, that in turn reduces the expression of Tissue Inhibitor of MetalloProteinases-3 (*TIMP3*) gene [168]. Moreover, it has been reported that *ATP2B1-AS1* over-expression in HRECs significantly reduces cell proliferation, migration, permeability, and angiogenesis induced by HG conditions, possibly by sponging miR-4729 and regulating the IQ motif-containing GTPase-activating protein 2 (IQGAP2) [169]. Furthermore, diabetic (vs. non-diabetic) mice display reduced expression of ***SOX2* Overlapping Transcript** (*SOX2OT*) [170]. Accordingly, primary retinal ganglion cells isolated from newborn mice and exposed to high-glucose or hydrogen peroxide display a marked reduction of *SOX2OT* levels in a time-dependent manner [170]. Notably, a transcriptomic analysis conducted on HRECs cultured in low glucose (LG), HG or HG *plus* transthyretin (HG + TTR) identified three new lncRNAs possibly associated with DR [171]. In particular, a strong upregulation of ***MSTRG.15047.3*** and ***AC008403.3*** has been observed, whereas a significant downregulation of ***FRMD6* Antisense RNA 2** (*FRMD6-AS2*) has been observed in LG and HG + TTR conditions compared to the cells treated with HG [171]. In line with these findings, humor aqueous and serum from DR patients display a significant downregulation of *FRMD6-AS2* [171]. Interestingly, the analysis of lncRNA-mRNA networks also suggested that *FRMD6-AS2* is likely to interact with *PBRM1* (Polybromo 1), *PPP2R5C* (Protein phosphatase 2 regulatory subunit B’gamma) and *ASB* (Arylsulfatase B), regulating cell proliferation and neovascularization [171]. Recently, the downregulation of two other lncRNAs has been observed in vitro in pathological contexts related to DR. Specifically, **miR-497 host gene** (*MIR497HG*) and **Transmembrane Phosphatase with Tensin homology Pseudogene 1** (*TPTEP1*) are strongly downregulated in HRECs and human retinal vascular endothelial cells (HRVECs) cultured in HG, respectively [172,173]. Notably, the overexpression of *MIR497HG* in HRECs and of *TPTEP1* in HRVECs significantly reduces cell proliferation and migration induced by HG treatment [172,173]. The phenotypic effects induced by *MIR497HG* are partially mediated by the binding of miRNA-128-3p that regulates *SIRT1* expression [172]. Moreover, it has been also demonstrated that *TPTEP1* reduces *VEGFA* levels by suppressing STAT3 phosphorylation and its nuclear translocation [173]. Additionally, in a recent paper it has been observed that **Mini-chromosome Maintenance Complex Component 3 Associated Protein Antisense** (*MCM3AP-AS1*) was downregulated in DR patients comparing to T2D subjects and promotes cell apoptosis by regulating miR-211/*SIRT1* axis [174]. Moreover, the downregulation of the **Long Intergenic Non-Coding RNA 673** (*LINC00673*) has been reported in plasma samples of DR patients [175]. Interestingly, *LINC00673* induces apoptosis in retinal pigment epithelial cells (RPECs) under HG conditions by negatively regulating p53. [175]. Interestingly, in a recent work from Sehgal and colleagues, the **Vascular Endothelial-Associated LncRNA 2** (*VEAL2*) has been identified as a novel lncRNA implicated in human vascular disease [176]. In particular, although an increase of *VEAL2* expression has been observed in the blood of DR patients, retinal choroid tissue of DR patients and HG-treated HUVECs display a reduced level of *VEAL2* [176]. Of note, *VEAL2* overexpression in HUVECs—cultured and not in HG—improves the excessive permeability phenotype by retaining the Protein kinase C beta (PRKCB2) in the cytoplasm, thus preventing translocation of junctional complexes from membrane to cytoplasm [176]. Furthermore, contrasting results have been observed for **BRAF-Activated Non-Protein Coding RNA** (*BANCR*). Particularly, follow-up studies reported both a decrease [177] and an increase [178] of *BANCR* expression in the plasma of patients with DR, compared both to patients without DR and healthy subjects [177]. Moreover, Zhang and colleagues also reported that *BANCR* overexpression inhibits the HG-induced apoptosis of ARPE-19 cells [177], while an opposite effect was shown by Yin and colleagues [178], indicating that further analyses are needed to clarify these controversies and to assess *BANCR* deregulation in hyperglycemia and DR-related contexts. Finally, a putative association between DR susceptibility and the deregulation of lncRNAs was suggested by genome-wide association studies (GWAS) in Japanese patients with T2D for the intergenic *RP1-90L14.1.* This lncRNA, also called **Long Intergenic Non-Coding RNA 1611** (*LINC01611*) is located on the chromosome 6q14.3, adjacent to the centrosomal protein 162 (*CE162*) gene which is involved in ciliogenesis [179]. Due to its proximity to *CE162* gene, the hypothesis is that this lncRNA could induce dysregulation in ciliary function by playing a role in susceptibility to DR [179].

All lncRNAs whose expression is decreased in DR and related contexts (Figure 1)—or for which contrasting results have been reported—are listed in Table 2, also including the main results discussed here.

## 4. The Deregulation of Small Nucleolar RNA Host Genes in Diabetic Retinopathy

An emerging class of lncRNA consists of the small nucleolar RNA host genes (SNHGs) that “host” small nucleolar RNAs (snoRNAs) in their introns. SnoRNAs are small RNAs of 60 to 300 bp located mainly in the nucleolus and primarily functioning as guide RNAs for post-transcriptional modifications of ribosomal and spliceosome RNAs [180] or involved in the post-transcriptional processing and maturation of ribosomal RNAs [181]. To date, 22 members of the SNHG family have been identified and involved in different cancer types, where they regulate cell proliferation, apoptosis, invasion, and migration [182]. Notably, five SNHGs have been reported as deregulated in DR, of which two have been described as upregulated and three as downregulated (Figure 1, Table 3).

Particularly, the Small Nucleolar RNA Host Gene 1 *(SNHG1)* is located on chromosome 11q12.3 region and hosts *SNORD31, SNORD28, SNORD29, SNORD26, SNORD27, SNORD30, SNORD22* and *SNORD25* [183]. *SNHG1* is upregulated in ARPE-19 exposed to HG conditions and its knockdown induces the reduction of vimentin, smooth muscle alpha-actin (α-SMA), IL-6 and IL-1β [184]. Moreover, *SNHG1* can induce the expression of E-cadherin and zonula occludens-1 (ZO-1), inhibiting migration and proliferation, and promoting cell apoptosis [184].

**Table 3:** Small nucleolar RNA host genes deregulated in diabetic retinopathy.

LncRNA	Chr	DR-Related Processes	Sponged miRNAs	Related Genes/Proteins	Ref.
*SNHG1*	11q12.3	Inflammation and apoptosis.		Vimentin, α-SMA, IL-6, IL-1β, E-cadherin, ZO-1	[184]
*SNHG16*	17q.25.1	Proliferation, migration, angiogenesis, apoptosis, oxidative stressand vessel-like formation.	miR-195, miR-146a-5p, miR-7-5p, miR-20a-5p	IRAK1, IRS1, NF-kB, PI3K/AKT, E2F1, mfn2	[185,186,187]
*GAS5 (SNHG2)*	1q25.1	Apoptosis, oxidative stress and inflammation.		BCL2, BAD, BACX, SERCA2b	[188,189]
*SNHG4*	5q31.2	Apoptosis.	miR-200b	OXR1	[190]
*SNHG5*	6q14.3	Cell proliferation and angiogenesis.		VEGFA	[191]
*SNHG7*	9q34.3	Proliferation, migration and angiogenesis.	miR-543, miR-34a-5p	SIRT1	[192,193]

Moreover, the **Small Nucleolar RNA Host Gene 16 (*SNHG16*)**—located on 17q.25.1 region and hosting *SNORD1A*, *SNORD1B* and *SNORD1C* [183]—could facilitate proliferation, migration, and angiogenesis in HRMECs cultured in HG and interact with miR-146a-5p and miR-7-5p, thus acting as ceRNA and affecting interleukin-1 receptor-associated kinase 1 (*IRAK1*) expression, as well as the substrate of the insulin receptor 1 (*IRS1*) [185]. Moreover, *SNHG16* overexpression has been associated with enhanced transactivation levels of NF-κB and forkhead box O (FOXO). Also, *SNHG16* overexpression positively regulates the PI3K/AKT pathway [185]. Notably, *SNHG16* was reported to be increased in proliferative DR compared to non-proliferative DR and healthy individuals [186], whereas an opposite trend was observed for miR-20a-5p, which is able to interact with *SNHG16* and E2F transcription factor 1 (*E2F1*). In line with these observations, *SNHG16* overexpression increases apoptosis and vessel-like formation, whereas miR-20a-5p partially reverses these effects [186]. Contrastingly, *SNHG16* was also reported as downregulated in HMRECs exposed to HG, AGEs, or hydrogen peroxide [187]. In addition, Zhang and colleagues demonstrated that over-expression of SNHG16 in HMRECs improves H_2_O_2_-induced angiogenesis by regulating miR-195/mitofusin 2 (mfn2) axis [187].

Additionally, the **Small Nucleolar RNA Host Gene 2 *(SNHG2)***—also known as Growth Arrest Specific 5 (*GAS5*)—is located within 1q25.1 region and contains 11 introns hosting different snoRNAs (*SNORD44*, *SNORD47*, *SNORD76*, *SNORD78*, *SNORD79*, *SNORD80*, *SNORD81*, *SNORD74*, *SNORD75* and *SNORD77*) [183]. *GAS5* encoded for a mRNA containing a small open reading frame (ORF) followed by some stop codons and multiple binding sites for nuclear hormone receptors, such as the glucocorticoid receptors [183]. The expression of *GAS5* is reduced in T2D patients with endometrial cancer [188] and its over-expression induces *BCL2*, also reducing the expression of the pro-apoptotic proteins BCL2 associated agonist of cell death (*BAD*) and *BAX* [189]. Moreover, it has been shown that in ARPE-19 cells exposed to HG, *GAS5* inhibits apoptosis and stress-induced inflammation of the endoplasmic reticulum by regulating sarcoplasmic/endoplasmic reticulum Ca2^+^ ATPase 2 (*SERCA2b*) [189].

Similarly, also the **Small Nucleolar RNA Host Gene 4 *(SNHG4)***—located in chromosomal 5q31.2 region and hosting *SNORD74A* and *SNORD74* [183]—is downregulated in DR patients and, in ARPE-19 cells grown in HG conditions, it suppresses cell apoptosis and regulates oxidation resistance protein 1 (OXR1) by sponging miR-200b [190].

Notably, the **Small Nucleolar RNA Host Gene 5 *(SNHG5)*** has been correlated to the development of diabetic macular edema (DME). This SNHG is located on chromosome 6q14.3 and hosts *SNORD50A* and *SNORD50B* [183]. The levels of *SNHG5* are impaired in the atrial fluid and blood of DME subjects, as well as in patients with refractory DME vs. those with idiopathic macular hole [191]. Moreover, *SNHG5* expression in plasma and aqueous humor negatively correlates with the duration of disease and body mass index [191]. Moreover, HRMECs exposed to HG display reduced expression of *SNHG5* [191]. Notably, *SNHG5* overexpression directly induce the downregulation of VEGF-a protein levels, decreasing cell proliferation and angiogenesis [191].

Finally, the **Small Nucleolar RNA Host Gene 7 *(SNHG7)***—located on chromosome 9q34.3 region and hosting SNORD17 and SNORD43 [183]—is also related to DR. Indeed, *SNHG7* negatively regulates miR-543 under HG conditions and is able to induce *SIRT1*. Notably, the activation of *SIRT1*/mir-543 pathway inhibit HG-induced cell proliferation, migration, and angiogenesis [192]. Moreover, *SNHG7* acts as ceRNA sponging miR-34a-5p [193]. Notably, *SNHG7* over-expression in HG-induced HRMECs represses EMT and tube formation through miR-34a-5p/X-box binding protein 1 (XBP1) pathway and the overexpression of miR-34a-5p is likely to revert this effect [193].

The list and main related results of small nucleolar RNA host genes SNHG deregulated in DR and related contexts are reported in Figure 1 and Table 3.

## 5. Conclusions and Perspective

Diabetic retinopathy is one of the most devastating complications of diabetes, both in terms of progression and permanent effects on patients. The molecular mechanisms underlying this microvascular dysfunction are yet to be fully elucidated. In recent years, increasing evidence shows that, in addition to well-known pathogenic mechanisms, epigenetic mechanisms could be at the basis of gene deregulation, which can underlie the alteration of key processes related to DR onset and progression. Notably, the permanent epigenetic modifications triggered by chronic hyperglycemia could be one of the key mechanisms underlying metabolic memory and the involvement of epigenetic factors, including the contribution of non-coding RNA, and need to be further addressed. For instance, among different classes of ncRNAs, the emerging role of lncRNAs in several cellular processes widely justify the research focus in multiple fields. Although many lncRNAs were initially studied in different types of cancer, accumulating evidence has also been indicating the pathogenic role of this class of ncRNAs in multifactorial diseases, including diabetes and its complications.

In this review, we systematically discussed the literature concerning the involvement of 50 lncRNAs in DR-related contexts. Some studies have been performed in patients with proliferative and non-proliferative DR, whereas several analyses have been performed using in vivo cell models or primary cells or cell lines, such as retinal epithelial cells, endothelial and Müller cells, usually exposed to HG concentrations or treated with different *stimuli* inducing oxidative stress. Although these models can only partially recapitulate the epigenetic and transcriptional deregulation underlying pathogenic mechanisms of DR, their use has been instrumental in revealing the putative role and the mechanism of action of several lncRNAs in DR, with particular attention to the aetiology related to oxidative stress development. Notably, a role as endogenous competitors for miRNA has been reported for various lncRNAs in DR and related contexts, indicating that complex interaction networks between lncRNA, miRNA and mRNA can play key roles in sustaining retinal homeostasis. However, further analyses are needed to better address the molecular mechanisms underlying the role of several lncRNAs in DR. Notably, since biomarkers for the early detection of DR have not been yet reported, the study of lncRNAs acquires additional relevance in light of their diagnostic and prognostic potential, as already assessed for different cancer types. Thus, a plausible future *scenario* could depict lncRNAs both as biomarkers and therapeutic targets in diabetic retinopathy, as well as in other diabetes complications.

## Figures and Tables

**Table 1:** Long non-coding RNAs reported as upregulated in diabetic retinopathy.

LncRNA	Chr	DR-Related Processes	Sponged miRNAs	Related Genes/Proteins	Ref.
*MALAT1*	11q13.1	Cell proliferation, migration, tube formation, vascular permeability, retinal vascularization, pericytes loss, capillary degeneration, microvascular leakage, oxidative stress, inflammation	miR-124, miR-20b, miR-17-3p, miR-124-3p, miR-125b-5p, miR-200b-3p, miR-203a-3p, miR-205-5p, miR-378a-3p, miR-320a	CREB, p38 MAPK, PP2A, NRF2, KEAP1, TNF-α, IL-6, SAA3, GRP78, CHOP, PRC2 complex, VE-cadherin/β-catenin, CDH5, YAP1, VEGFA, HIF-1α, Pde6g, Guca1a, Rho, Sag, Prph2, MCP-1	[30,32,33,34,35,36,37,38,39,40,41,42,43,44,45,46,47,48,49,50,51,52,53,54,55,56,57,58]
*MIAT*	22q12.1	Cell proliferation, migration, tube formation, cell viability, apoptosis, microvascular permeability, vascular leakage, inflammation, pyroptosis	miR-150-5p, miR-29b, miR-342–3p	Casp3, AKT1, VEGF, NF-κB, IL-6, IL1B, TXNIP, CASP1, TGFB1	[59,60,61,62,63,64,65,66,67,68,69,70,71,72]
*ANRIL*	9p21.3	Regulates VEGF expression, inflammation, apoptosis	miR-200b	VEGF, Bax, P65, Bcl-2, IL-1, IL-10, MCP-1, p300, EZH2 (PRC2 complex)	[62,82,83,84]
*NEAT1*	11q13.1	Cell proliferation, cell apoptosis, epithelial-mesenchymal transition, oxidative stress, inflammation	miR-497, miR-204	BCL2, BAX, SOX4, COX2, IL-6, TNF-α, VEGFA, TGF-β1, BDNF	[58,87,88,89]
*HOTAIR*	12q13.13	Angiogenesis, oxidative damage, mitochondrial alterations, retinal acellular capillaries, vascular leakage, proliferation, invasion, migration, permeability	miR-20b, miR-17-3p	LSD1, VE-cadherin, H3K4me3, HIF1α, VEGFA	[52,92,93]
*KCNQ1OT1*	11p15.5	Proliferation, apotosis, epithelial-mesenchymal transition	miR-214, miR-1470	CASP1, EGFR, IL-1β	[98,99,100]
*HEIH*	5q35.3	Apoptosis and cell damage	miR-939	VEGF, PI3K/AKT1, CASP3, CYPC	[101,102]
*RNCR3*	8p23.1	Retinal vascular functions, cytokines release	miR-185-5p	Klf2	[103,104,105]
*HOTTIP*	7p15.2	Cell viability, apoptosis		VEGF, ICAM-1, P38/MAPK	[107]
*BDNF-AS*	11p14.1	Apoptosis		BDNF	[108,109,110]
*FENDRR*	16q24.1	Proliferation, angiogenesis		FOX1, VEGF	[111,112,113]
*IGF2AS*	11p15.5	Apoptosis		IGF2/AKT, CASP-9	[114]
*Arid2-IR*	12q12	inflammation, oxidative stress, apoptosis		Smad-3, Bax, Bcl2	[115]
*TDRG1*	6p21.2	Proliferation, permeability, migration, tube formation		VEGF	[116]
*AQP4-AS1*	18q11.2	Retinal neurodegeneration, capillary degeneration		AQP4	[117]
*LINC00323*	21q22.2	Proliferation, angiogenesis		GATA2	[118]
*MIR503HG*	Xq26.3	Proliferation, angiogenesis.		GATA2	[118]
*HIF1A-AS2*	14q23.2	Hypoxia, oxidative stress, angiogenesis		HIFα, VEGF, MAPK	[119]
*LINC00174*	7q11.21	Proliferation, migration, angiogenesis	miR-150-5p	VEGFA	[120]
*TUG1*	22q12.2	Proliferation, migration, tube formation	miR-145, miR-524-5p	VEGFA, FGFR2	[121,124]
*UCA1*	19p13.12	Epithelial-mesenchymal transition	miR-624-3p	VEGFC	[122]
*LINC00963*	9q34.11	Proliferation, invasion, migration	miR-27b		[123]
*PVT1*	8q24.21	Proliferation, migration	miR-128-3p		[62,125]
*OGRU*	chr9qA4	Inflammation, oxidative stress	miR-320	TGF-β1, USP14	[126]

**Table 2:** Long non-coding RNAs reported as s downregulated in diabetic retinopathy.

LncRNA	Chr	DR-Related Processes	Sponged miRNAs	Related Genes/Proteins	Ref.
*MEG3*	14q32.2	Proliferation, migration, angiogenesis, oxidative stress, inflammation, neovascolarization	miR-34a, miR-223-3p, miR-204, miR-93, miR-19b, miR-6720-5p	NF-kB, DNMT1, PI3K, Akt, mTOR, IL-1β, VEGF, NRF2, SOCS6, CYB5R2, Sirt1, Notch1	[129,130,131,132,133,134,135,136,137,138,139,140]
*XIST*	Xq13.2	Apoptosis, migration, inflammation	miR-21-5p	VEGF, SIRT1	[146,147]
*H19*	11p15.5	Endothelial–mesenchymal transition, vascular leakage, inflammation	miR-675-3p, miR-675-5p, miR-200b, miR-93, miR-19b	XBP1, SIRT1, TGF-β1, Smad	[17,149,150,151,152,153,154,155,156,157,158]
*LINC-PINT*	7q32.3	Cell viability		P53	[159,160]
*VIM-AS1*	10p13	Apoptosis	miR-29		[161]
*LUADT1*	6q24.3	Apoptosis	miR-383	PRX3	[166]
*AK077216*	8p23.2	Apoptosis	miR-383		[167]
*RPSAP52*	12q14.3	Apoptosis	miR-365	TIMP3	[168]
*ATP2B1-AS1*	12q21.33	Proliferation, migration, angiogenesis, permeability	miR-4729	IQGAP2	[167,169]
*SOX2OT*	3q26.33	Retinal neurodegeneration			[170]
*FRMD6-AS2*	14q22.1	Proliferation, neovascularization		PBRM1, PPP2R5C, ASB	[171]
*MIR497HG*	17p13.1	Proliferation, migration	miR-128-3p	SIRT1	[172,173]
*TPTEP1*	22q.11.1	Proliferation, migration		STAT3, VEGFA	[173]
*MCM3AP-AS1*	21q22.3	Apoptosis	miR-211	SIRT1	[174]
*LINC00673*	17q24.3	Regulation of P53, apoptosis		P53	[175]
*VEAL2*	16p12.2	Endothelial permeability		PRKCB	[176]
*BANCR*	9q21.11-q21.12	Apoptosis			[177,178]
*RP1-90L14.1*	6q14.3	Ciliary function		CE162	[179]

## Data Availability

Not applicable.
